# Editorial: Intracellular Proteasome Dynamics

**DOI:** 10.3389/fmolb.2020.00143

**Published:** 2020-07-17

**Authors:** Cordula Enenkel, Richard S. Marshall, Richard D. Vierstra

**Affiliations:** ^1^Department of Biochemistry, University of Toronto, Toronto, ON, Canada; ^2^Department of Biology, Washington University in St. Louis, St. Louis, MO, United States

**Keywords:** ubiquitin-proteasome system, proteasome localization, protein homeostasis, protein degradation, proteasome granules

In eukaryotes, proteasomes are abundant proteolytic complexes that direct the degradation of numerous short-lived proteins whose function is required within a limited time frame, or proteins whose structure is damaged. If these unwanted proteins are not cleared by proteasomes, cells become susceptible to challenging environmental conditions such as nutrient starvation and physical or chemical stress. Failure to overcome these challenges can accelerate aging and cause diseases such as cancer and neurodegeneration, thus providing strong medical relevance to proteasome research.

To degrade proteins at the right time and in the right place, proteasomes must be highly dynamic, not only with regard to their intracellular localization and subunit composition, but also in their specificity for particular substrates, which permits them to serve diverse protein degradation pathways. All proteasomes contain the proteolytic core complex, named the 20S core protease (CP), which can interact with different individual regulatory proteins or multi-subunit regulatory complexes, the most common of which is known as the 19S regulatory particle (RP) that caps one or both ends of the CP. This 26S CP-RP holo-complex is composed of at least 33 different subunits, along with numerous accessory proteins that transiently interact with the particle to help identify appropriate substrates and ensure that proteasomal degradation is tightly regulated, as reviewed by Kors et al..

Proteasome substrates are often targeted for degradation by the covalent attachment of poly-ubiquitin chains, which are recognized by intrinsic and extrinsic ubiquitin receptors associated with the proteasomal regulatory complex. In bacteria, a ubiquitylation-like event termed pupylation exists as an archaic degradation signal that allows specific substrates to access proteasome-like proteases, indicating that the delivery of substrates into proteasomes is conserved in prokaryotes and eukaryotes. Deubiquitylation or depupylation peptidase activities associated with regulatory subunits of the proteasome typically release this signal prior to substrate degradation so it can be reused. Disordered or unstructured protein substrates can additionally be degraded without being first ubiquitylated or pupylated, confirming that proteasomes possess a dynamic interactome with a diversity of substrates, as outlined by Müller and Weber-Ban.

Since the proteolytic active sites that cleave potentially toxic substrates into harmless peptides are secluded inside the CP cavity, proteasomes are essentially a self-compartmentalized garbage disposal. This self-compartmentalization begins during proteasome maturation, as subcomplexes are assembled from inactive precursors bearing inhibitory propeptides that must be auto-catalytically cleaved within the nascent proteolytic cavity to fully complete CP assembly, as reviewed by Marshall and Vierstra. Precise regulation of proteasomal gene expression is also crucial for both the assembly and dynamic rearrangement of proteasome complexes. The transcription factors Rpn4 (in yeast) and NRF1 (in mammals) augment proteasomal gene expression in response to proteasomal dysfunction and nutrient-induced mTORC (mammalian target of rapamycin complex I) activation, while mammalian NRF2 performs this role in response to oxidative stress. The regulation of proteasomal gene expression on a post-transcriptional level through MAP (mitogen-activated protein) kinases facilitates subunit incorporation during proteasome assembly, as reviewed by Motosugi and Murata. A plethora of post-translational modifications of the 26S complex then also modify proteasome activities, influence its intracellular localization, and ultimately help trigger its turnover (Kors et al.; Marshall and Vierstra).

Due to the intricate assembly mechanism of proteasomes, the labeling of a proteasomal subunit with fluorescent reporters such as green fluorescent protein (GFP) can be problematic, and thus should be used as a reliable reporter of the active holo-enzyme only if the GFP fusion protein is fully incorporated into the mature proteasome complex. Since proteasome subunits also exist as part of inactive precursor complexes, activity-based probes have been developed to specifically target the active sites of the CP. These probes penetrate the plasma membrane of mammalian cells and illuminate active proteasomes, thus permitting their localization. In combination with either indirect fluorescence microscopy using proteasome-specific antibodies or direct fluorescence microcopy using GFP-labeling techniques, the mature CP can now be localized with high fidelity, as shown by Schipper-Krom et al.. For example, using activity-based probes mimicking proteasomal peptides, a prominent nuclear localization of the CP was apparent within the mammalian cancer cell line MelJuSo, as shown by Gan et al.. To co-localize activity-based probes with GFP-labeled proteasomal subunits, the immuno-proteasome CP subunit β5i is an ideal recipient for the GFP fusion, as its incorporation into the CP is highly efficient upon release of cytokines. Almost 90% of the GFP-labeled immuno-CP is nuclear in U2OS and Hela cells. After photobleaching of the entire cytoplasm, proteasomes remain in the nucleoplasm, indicating the presence of large holo-proteasome complexes (Schipper-Krom et al.).

In proliferating yeast cells, GFP labeled proteasomes are also mainly nuclear, an observation independent of which CP or RP subunit is fused with the GFP reporter protein. These observations provide evidence that proteasome localization is highly conserved in eukaryotes. Studies in yeast have also provided insights into the intracellular movement of proteasomes, which are imported into the nucleus as either immature precursor complexes or mature holo-enzymes, depending on their availabilities under different growth conditions. The nuclear import of proteasome complexes uses the classical import receptor importin/karyopherin αβ, together with transiently associated proteins to either assist in proteasome assembly or confer classical nuclear localization signals to mature proteasomes. Proteasome-associated regulatory proteins such as yeast Blm10 (PA200 in mammals) also facilitate nuclear localization of the CP, as summarized by Wendler and Enenkel.

Upon nutrient starvation, proteasomes surprisingly exit the nucleus into the cytoplasm. When yeast cells are deprived of glucose, decreasing energy sources [in the form of reduced adenosine triphosphate (ATP) levels] and acidosis cause them to transition into quiescence and sequester proteasomes into motile and reversible cytoplasmic condensates known as proteasome storage granules (PSGs). These membraneless PSGs are considered to serve as sorting compartments in which functional proteasomes are saved and separated from non-functional proteasomes as summarized by Karmon and Ben-Aroya. Functional proteasomes within proteasome storage granules are highly mobile and remain ready for reimport back into the nucleus upon exit from quiescence and the resumption of growth (Wendler and Enenkel).

Environmental challenges leading to proteasome inhibition also induce the spatial reorganization of proteasomes into nucleoplasmic or cytoplasmic aggregates (Karmon and Ben-Aroya). A sophisticated sorting mechanism seems to distinguish active and functional proteasome complexes from their inactive and non-functional counterparts. Comprehensive studies in yeast, plants, and mammalian cells have revealed that inactive and non-functional proteasomes are first ubiquitylated and sequestered by proteasome-specific receptors for autophagic elimination, while active and functional proteasomes are protected from vacuolar or lysosomal degradation in PSGs (Marshall and Vierstra). New therapeutic schemes can thus be envisioned to enhance proteasome rehabilitation.

The life cycle of proteasome homeostasis, starting with regulated proteasomal gene expression, proteasome assembly into interchangeable proteasome complexes with different configurations, altered proteasome localization in response to changing growth conditions and environmental challenges, and finally autophagic turnover of excess or inactive proteasomes offer interesting starting points for therapeutic interventions. The significance of future work on proteasome dynamics can be envisioned for age-related proteasome movements in mammalian cells in which old proteasomes are cytoplasmic, while newly produced proteasomes are primarily nuclear. Differences in the localization and proteolytic capacity of proteasomes may have significant impact on protein homeostasis in health and disease.

## Author Contributions

All authors listed have made a substantial, direct and intellectual contribution to the work, and approved it for publication.

## Conflict of Interest

The authors declare that the research was conducted in the absence of any commercial or financial relationships that could be construed as a potential conflict of interest.

